# Supplementary material for: A fault-intrusion-tolerant system and deadline-aware algorithm for scheduling scientific workflow in the cloud
Source: PeerJ Comput Sci. 2021 Nov 2;7:e747. doi: 10.7717/peerj-cs.747 (PMC8576554; doi:10.7717/peerj-cs.747)
Supplement: Supplemental Information 6 [file peerj-cs-07-747-s006.zip › Myproject1.1/docs/org/cloudbus/cloudsim/power/models/package-frame.html]

org.cloudbus.cloudsim.power.models


# org.cloudbus.cloudsim.power.models

## Interfaces

- *PowerModel*

## Classes

- PowerModelCubic
- PowerModelLinear
- PowerModelSpecPower
- PowerModelSpecPowerHpProLiantMl110G3PentiumD930
- PowerModelSpecPowerHpProLiantMl110G4Xeon3040
- PowerModelSpecPowerHpProLiantMl110G5Xeon3075
- PowerModelSpecPowerIbmX3250XeonX3470
- PowerModelSpecPowerIbmX3250XeonX3480
- PowerModelSpecPowerIbmX3550XeonX5670
- PowerModelSpecPowerIbmX3550XeonX5675
- PowerModelSqrt
- PowerModelSquare
